# Effects of Multivitamin, Multimineral and Phytonutrient Supplementation on Nutrient Status and Biomarkers of Heart Health Risk in a Russian Population: A Randomized, Double Blind, Placebo Controlled Study

**DOI:** 10.3390/nu10020120

**Published:** 2018-01-25

**Authors:** Vasily A. Isakov, Alexandra A. Bogdanova, Vladimir V. Bessonov, Tatiana B. Sentsova, Victor A. Tutelyan, Yumei Lin, Valentina Kazlova, Jina Hong, Rodney A. Velliquette

**Affiliations:** 1 Institute of Nutrition, Ustinsky Proezd, 2/14, Moscow 109240, Russia; alex.golubeva@gmail.com (A.A.B.); bessonov@ion.ru (V.V.B.); bio45@inbox.ru (T.B.S.); tutelyan@ion.ru (V.A.T.); 2 Access Business Group International, LLC, 5600 Beach Blvd., Buena Park, CA 90621, USA; ylin@yumeiconsulting.com (Y.L.); valentina.kazlova@amway.com (V.K.); rod.velliquette@amway.com (R.A.V.)

**Keywords:** heart health risk, homocysteine, nutrient status, dietary supplementation, quercetin, multivitamin, multimineral, phytonutrient

## Abstract

The primary objective of this clinical study was to evaluate the effect of a dietary multivitamin, multimineral and phytonutrient (VMP) supplement on blood nutrient status and biomarkers of heart health risk in a Russian population. One hundred twenty healthy adults (40–70 years) were recruited for a 56-day (eight-week) randomized, double blind, placebo controlled study with parallel design. Subjects were divided into two groups and received either a VMP or a placebo (PLA) supplement. Blood nutrient levels of β-carotene, α-tocopherol, vitamin C, B6, B12, red blood cell (RBC) folate, Zinc and Selenium were measured at baseline and on Days 28 and 56, and quercetin was measured at baseline and on Day 56. Blood biomarkers of heart health, i.e. homocysteine (Hcy), high-sensitivity C-reactive protein (hs-CRP), oxidized LDL (ox-LDL), gamma-glutamyl transferase (GGT), uric acid and blood lipid profile, were measured at baseline and Day 56. Dietary VMP supplementation for 56 days significantly increased circulating levels of quercetin, vitamin C, RBC folate and partially prevented the decline in vitamin B6 and B12 status. Both serum Hcy and GGT were significantly reduced (−3.97 ± 10.09 µmol/L; −1.68 ± 14.53 U/L, respectively) after VMP supplementation compared to baseline. Dietary VMP supplementation improved the nutrient status and reduced biomarkers of heart health risk in a Russian population.

## 1. Introduction

A fruit and vegetable rich diet is associated with reduced risk for chronic diseases such as cardiovascular disease (CVD) [1]. Fruits and vegetables are natural source of vitamins, minerals and phytonutrients (phytochemicals, which have a positive effect on human health), and some of these components have been reported to have antioxidant and cardioprotective effects in clinical trials [2,3,4]. However, randomized, double blind, placebo controlled trials examining whether phytonutrients have protective effects against the development of CVD are still lacking.

A poor antioxidant defense status can promote the generation of free radicals, which have been reported to be positively correlated with blood homocysteine (Hcy) levels [5,6]. Elevated Hcy concentration is thought to be a risk factor for CVD and may accelerate the progression and the initial incidence of arteriosclerosis via free radical generation [7]. In addition to antioxidant intake and defense status, dietary folate, vitamin B6 and vitamin B12 intake, gender and age can impact blood Hcy levels [8,9,10]. Multiple lifestyle behaviors also influence chronic disease development. Studies have shown that lower serum antioxidants levels are present in smokers compared to non-smokers [11,12]. Alcohol consumption is high in Russian population and low levels of antioxidants have been found in chronic drinkers and evidence suggests an inverse relationship between antioxidant status and CVD [13].

Data from the Moscow Behavioral Risk Factor Survey (2000–2001) indicated the mean fruit and vegetable intake in men and women in Moscow was much lower (190 g/day) than the World Health Organization’s recommended 400 g/day [14,15]. The World Health Survey (2002–2003) data indicated that approximately 80% of Russians consumed lower than the WHO recommended levels [16]. Using data from the Health in Times of Transition data set (2010), Goryakin et al. reported that only 42.7% of Russia adults consumed fruits and vegetables daily or several times weekly [17]. Together, these data strongly suggest that Russian adults consume far below the World Health Organization (WHO)’s recommendation for fruits and vegetable intake. This lower than recommended intake would likely lead to lower intake of vitamins, minerals and phytonutrients, which could lead to higher risks for chronic diseases such as CVD [18]. Therefore, promoting healthy dietary and lifestyles behaviors, and supplementing for deficiencies becomes a critical approach in preventing chronic disease development.

In this clinical study, we examined whether a multivitamin, multimineral and phytonutrient product could improve the nutritional status and reduce biomarkers of heart health risk in a Russian population with low fruit and vegetable intake. The primary outcome of the study was to evaluate the effect of a multivitamin, multimineral and phytonutrient (VMP) product on nutrient status and biomarkers of heart health risk factors; and secondary outcome was to test the safety and tolerability of a VMP product.

## 2. Materials and Methods

### 2.1. Study Population and Design

**Figure 1:**
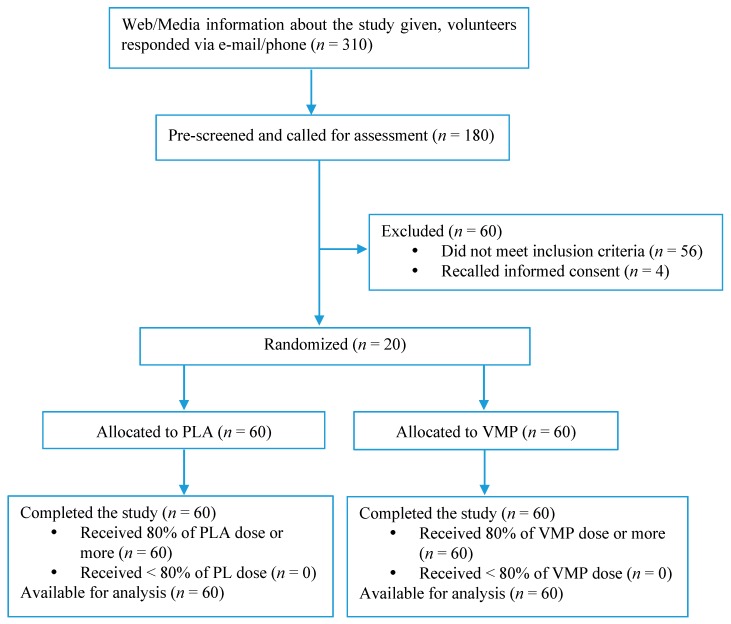
Consort diagram summarizing the subjects disposition for the study.

The study was 56-day (eight-week) randomized, double blind, placebo controlled clinical trial. One hundred twenty generally healthy (without chronic diseases) adults (40–70 years) from Moscow, Russia were recruited. Subjects had habitually low dietary intake of recommended foods, predominantly fruit and vegetable, as classified by a recommended food score (RFS) of <12 (score range, 0–23). The RFS is based on consumption of recommended foods in a 62-item Food Frequency Questionnaire (FFQ) with modifications according to availability of food items in the Russia area [19] (see Appendix A for list of foods). Subjects were randomized to either receive a placebo (PLA) (*n* = 60) or VMP (*n* = 60) supplement (Figure 1). The study was conducted at the Institute of Nutrition of Russian Academy of Medical Sciences and followed the Good Clinical Practice guidelines and the Declaration of Helsinki (1996). The study was approved by the Institute of Nutrition IRB (Moscow, Russia) according to Good Clinical Practice and Helsinki Declaration (Sixth revision, 2008). Table 1 shows the inclusion and exclusion criteria for subjects, and Table 2 illustrates the summary of study visits and procedures. This study was registered at ClinicalTrials.gov (NCT02224092).

### 2.2. Study Samples

There were two study samples: (1) a VMP; and (2) a PLA. Each sample contained three different types of tablets; (1) a vitamin; (2) a mineral; and (3) a phytonutrient. The VMP sample (six Nutrilite Double X™ tablets) provides the following micronutrients daily: 14 vitamins (800 μg Vit A, 5.0 μg Vit D, 16 mg Vit E, 55 μg Vit K, 2.0 mg Vit B1, 2.4 mg Vit B2, 3.0 mg Vit B6, 2.0 μg Vit B12, 180 mg Vit C, 26 mg niacin, 60 μg biotin, 3.0 mg β-carotene, 400 μg folate and 10 mg pantothenic acid), nine minerals (471 mg calcium, 5.0 mg iron, 37.5 μg iodine, 6 mg zinc, 0.75 mg copper, 1 mg manganese, 55 mg magnesium, 25 μg chromium and 50 μg selenium) and the phytonutrient tablet contained apple, bilberry, grape seeds, plum, pomegranate, cranberry juice, grape and rosemary extracts, and parsley, carrot, broccoli, spinach and horseradish powders. The PLA sample for this trial was formulated to match the shape and color of all VMP sample tablets, e.g., a vitamin placebo, a mineral placebo and a phytonutrient placebo. PLA sample contained dextrose (or maltodextrin), microcrystalline cellulose, croscarmellose sodium and magnesium stearate. All tablets were manufactured in a Good Manufacture Practice certified facility, Access Business Group International, LLC (Buena Park, CA, USA).

Subjects were instructed to consume study samples twice a day, once in the morning and once in evening with meal (6 tablets/day). To ensure compliance, each serving was packed in an individual sachet and a monthly supply was provided to participants. The directions for consumption of the different study samples were printed on the bag. Compliance was assessed by counting the number of tablets at each visit and patients were considered compliant if they took 80% or more of total number of VMP or PLA samples. Blood samples were collected from all subjects during office visits at baseline (Day 1 prior to consuming study samples), Day 28 and Day 56. All samples were stored at −80 °C until analysis.

### 2.3. Questionnaires

Dietary macro- and micronutrients intake were determined using a PC-based FFQ (FFQ-1.0, Institute of Nutrition, Moscow, Russia) validated earlier in patients with different diseases as well as healthy Russian population [20]. A questionnaire on medical history, family medical history, smoking habits and history, alcohol consumption habits and history, exercise habits and dietary supplement use (current and history) was also administered to all subjects. 

### 2.4. Nutrient Analysis

Blood samples were taken in fasting condition according to protocol (Table 2). Serum β-carotene and total tocopherols (alpha- and gammatocopherol) were assessed simultaneously by isocratic rapid High Performance Liquid Chromatography (HPLC) [21]. In brief, 200 μL of serum was deproteinated by the addition of 200 μL ethyl alcohol containing 0.5 μmol/L echinenone and 4 μmol/L tocol with 30 mg/L butylated hydroxytoluen. The samples were vortexed for 2 min, followed by addition of 800 μL hexane and centrifuged for 10 min at 1500 rpm. The hexane extraction process was repeated, and the supernatants were combined and evaporated under nitrogen. The residues were dissolved in 100 μL mobile phase, containing 30 mg/L BHT and 20 μL was injected into an Agilent 1100 HPLC system (Agilent Technologies, Waldbronn, Germany) equipped with diode array detector (DAD) and fluorescence detector (FLD). A Javelin guard column (3 mm × 20 mm, Spherisorb ODS2 3 µm (C18)) connected to Thermo Spherisorb ODS2 column (3 mm × 250 mm, 3 µm) (Fisher Scientific UK Ltd., Loughborough, UK) was used. The DAD detector was adjusted at 455 nm for the β-carotene. FLD detector was configured at λex = 290 nm and λem = 335 nm for the tocopherols. Standards of β-carotene, and tocopherols were obtained from Sigma-Aldrich (St. Louis, MO, USA). 

Plasma vitamin (Vit) C, B6, B12 and red blood cells (RBC) folate measurement were performed using enzyme immunoassay (EIA) kits according to manufacturer instructions (BCM Diagnostics, Moscow, Russia). Total plasma selenium was determined by simplified fluorometric assay [22]. Serum zinc level was assessed by indirect colorimetry using QuantiChrom™ Zinc kit per manufacturer instructions (BioAssay Systems, Hayward, CA, USA). Blood calcium, magnesium and iron were assessed by turbidimetry using automated analyzer Konelab 30i (Thermo Fisher Scientific Messtechnik GmbH, Oberhausen, Germany). Plasma quercetin concentration was measured by HPLC/MS (Agilent 1200; Mass Spectrometer: Agilent Triple Quad 6410, Agilent Technologies, Waldbronn, Germany) after treatment with a beta-glucuronidase/sulfatase mixture and extraction into ethylacetate as described previously [23].

### 2.5. Biomarkers of Heart Health Risk

Blood total cholesterol, high-density lipoprotein (HDL), low-density lipoprotein (LDL), gamma-glutamyl transferase (GGT), and triglycerides were assessed by turbidimetry using automated analyzer Konelab 30i (Thermo Fisher Scientific Messtechnik GmbH, Oberhausen, Germany). Total serum homocysteine level was assessed by using Axis Homocysteine EIA kit (Axis-Shield Diagnostics Ltd., Dundee, UK). Briefly, protein-bound homocysteine was reduced to free homocysteine and enzymatically converted to *S*-adenosyl-l-homocysteine in a separate procedure prior to the immunoassay, according to manufacturer instruction. Serum high sensitivity C-reactive protein (hs-CRP) was assessed by using EIA kit (Diagnostics Biochem Canada Inc., Dorchester, ON, Canada) per manufacturer instructions. Serum oxidized LDL (ox-LDL) concentration was measured with an ox-LDL EIA kit (Biomedica Medizinprodukte GmbH, Wien, Austria) per manufacturer instructions.

Safety and tolerability of study samples was assessed by measuring changes in serum chemistry, hematology, vital signs and body weight. Potential study sample related adverse events (AE) were assessed by a self-reported questionnaire during follow up phone interview (Table 2).

### 2.6. Data Analysis

Statistical analysis was done with Statistica 6.0 software by StatSoft (Tulsa, OK, USA). Graphical method (histograms) and non-parametrical test of Kolmogorov–Smirnov were used to verify the distribution of interval variables. Methods of parametric and non-parametric statistics were used based on the variables type and distribution and the number of controls. Student’s T-test and Mann–Whitney criteria were used to estimate the rate of differences between 2 independent selections. Student’s *T*-test and Wilcoxon’s criteria were used to estimate the rate of differences between 2 co-dependent selections. χ^2^ criteria or Fishers double-sided criteria was used to estimate reliability of quality parameters distribution. Benjamini–Hochberg procedure was used to control the False Discovery Rate (FDR) when performing multiple comparisons. Data are presented as mean value ± standard deviation except where dichotomized data is presented.

## 3. Results

Subjects were healthy, had no medical conditions or complaints, and were not receiving any medical treatment or consuming dietary supplements containing vitamins, minerals or herbals ingredients. All randomized subjects completed the protocol, no drop-outs were registered (Figure 1). Table 3 shows subjects baseline characteristics, lifestyle behaviors and biomarkers of heart health risk. Subjects were predominantly women, but there was no difference in gender between two studied groups. There were no significant differences between groups in any of the measurements, however, both groups on average were overweight and had elevated serum Hcy levels (>15 µmol/L). In addition, there were no significant differences in nutrient consumption and blood nutrient status between groups at baseline (Table 4). However, excess consumption of fats and sodium were indicated from FFQ in both groups.

Table 5 presents the effect of dietary supplementation with study samples on the change (delta) in blood nutrient status at Days 28 and 56 (baseline to Day 28 or 56). After 28 days of dietary VMP supplementation, RBC folate levels increased significantly from baseline and when compared to PLA (3.40 ± 5.88 µg/L and 0.71 ± 5.86 µg/L, respectively; *p* < 0.01). Serum Vit B6 levels decreased significantly from baseline at Day 28 in the PLA group and was significantly different than VMP group (−4.58 ± 6.48 µg/L and −0.02 ± 7.26 µg/L, respectively; *p* < 0.001). Serum Vit B12 decreased significantly from baseline in both groups at Day 28.

After 56 days of dietary VMP supplementation, Vit C levels increased significantly from baseline and when compared to PLA (8.21 ± 9.27 mg/dL and 2.97 ± 9.09 mg/dL, respectively; *p* < 0.01) (Table 5). The change in RBC folate levels remained elevated from baseline in VMP group at Day 56, while levels did not change in PLA group (3.20 ± 7.18 µg/L and −0.23 ± 6.01 µg/L, respectively, *p* < 0.003). Serum Vit B6 status continued to significantly decline from baseline in the PLA group (−6.83 ± 7.58 µg/L), which was not observed in VMP group (*p* = 0.001). The decline in Vit B12 status from baseline persisted at Day 56 in PLA group (−84 ± 204 ng/mL), while the VMP group seemed to partially recover from the initial declined seen at Day 28 (Table 5). Fifty-six days of dietary VMP supplementation significantly increased plasma quercetin compared to baseline, and when compared to PLA group (4.28 ± 8.58 ng/mL and −0.08 ± 9.45 ng/mL, respectively; *p* < 0.005).

Table 6 shows the change on heart health biomarkers after 56 days of dietary supplementation with study samples. Serum Hcy levels decreased significantly from baseline in the VMP group and when compared to the PLA group (−3.97 ± 10.09 µmol/L and −0.82 ± 8.16 µmol/L, respectively; *p* = 0.031). Serum GGT levels were significantly reduced from baseline only in the VMP group and tended be to different compared to PLA group (−1.68 ± 14.53 U/L and 1.99 ± 9.86 U/L, respectively; *p* = 0.054). There were no significant effects on the lipid profile after dietary supplementation with study samples in either group (Table 6). There were no changes in blood chemistry and hematology measurements, and no AE reported during the study.

## 4. Discussion

This is the first randomized, double blind placebo, controlled study investigating the effect of the vitamin-mineral complex with phytonutrients on nutrient status and biomarkers of heart health risk factors in a generally healthy adult Russian population. There were three main categories of outcome measures examined in the clinical study: (1) plasma levels of water-soluble antioxidants (i.e., Vit C and quercetin) and fat-soluble antioxidants (i.e., β-carotene); (2) circulating levels of the “anti-homocysteine triad”, Vit B6, Vit B12 and RBC folate; and (3) biomarkers associated with heart health such (i.e., serum Hcy and GGT). The key findings of this study were that a dietary VMP supplementation (Nutrilite Double X™) improved overall Vit B status and reduced serum Hcy levels compared to PLA group.

It was not surprising that, among randomized subjects, women were predominant, as, according to many studies, women practiced more healthy habits and had a higher dietary supplement intake rate than men [24,25,26]. Moreover, this gender difference in dietary supplement consumption is more prominent in younger age and disappears after six/seven decade of life [27]. All subjects included the study had low consumption of fruits and vegetables based on the modified Recommended Foods Checklist. This is in line with previously reported fruit and vegetable intake in this population, and is far from the 400 g/day recommended by WHO. Low consumption of fruits and vegetables is also recognized as CVD risk factor, and enriching the diet with fruits and vegetables is considered as one of the effective tools for CVD prophylaxis [28,29].

Low folate status is associated with an increased CVD risk [30,31]. One mechanism by which folate (and overall B vitamins) has been shown to be protective in the development of CVD is by reducing serum Hcy levels. B vitamins, specifically folate, B6 and B12 are essential for recycling of Hcy back to methionine or for cysteine synthesis. If Hcy is not properly recycled, elevated levels have been reported to cause an increased risk for developing CVD and other age-related diseases [32,33,34,35,36]. In healthy populations, the main nutritional cause of elevated blood Hcy is folate deficiency, which appears to be the case for the subjects in this study. Furthermore, low Vit B12 status has been reported to be a reliable surrogate marker of elevated Hcy [37]. The low overall B vitamin status that was observed in the PLA group during the 56 days trial is in line with previous reports on Russian urban population [38]. Dietary supplementation with folate and B vitamins or food fortification is a first line treatment for elevated Hcy levels, as at least 0.8 mg/day of folate is necessary to achieve the maximal reduction of Hcy [39]. Therefore, increasing folate (and overall B vitamins) status, similar to what was achieved in the VMP supplemented group, may translate into benefits of reducing CVD risk if subjected continued supplementation, but this needs to be proved in long-term prospective controlled trials.

Serum hs-CRP is considered a biomarker of CVD risk; however, levels were not significantly influenced by the VMP supplementation. In several population-based studies, blood level of hs-CRP correlated with blood pressure, age, blood glucose level and BMI [40,41], which are established CVD risk factors. In addition, a chronic, continuous elevation in hs-CRP has been reported to be a more relevant CVD risk than the high level found in one measurement [42]. hs-CRP is a marker of systemic inflammation (both acute and chronic), which can change in both directions depending on the inflammation course. Therefore, it is not surprising that during the short duration of our study we found high variability in hs-CRP levels and no significant changes after the dietary supplementation.

Free radicals or reactive oxygen species (ROS) are generated by normal energy metabolism and are controlled by antioxidant pathways. When there is insufficient removal of these ROS, the oxidative damage can increase the risk of CVD development [43,44]. Both Vit C and quercetin are known to be effective antioxidants. Quercetin is one of the most abundant flavonoid found in fruits, vegetables, leaves and grains, and is widely used as a dietary supplement in the food industry. Quercetin has been reported to modulation various of biological pathways, including cellular signaling events implicated in CVD development [45,46,47,48]. In a population that consumes low levels of fruits and vegetables, the higher plasma Vit C and quercetin levels found after the VMP supplementation could contribute to a lower risk of developing CVD if one was to chronically consume these antioxidants.

Vit C and polyphenols (flavonoids and non-flavonoids) are major natural antioxidants capable of preventing damage generated by oxidative stress [49]. It is possible that these benefits observed with VMP supplementation could be due to the combination of multiple vitamins, minerals and/or polyphenols, such as quercetin. Quercetin was reported to reduce Hcy by increasing remethylation and transsulfuration of homocysteine in rats [50]. Moreover, the combination of quercetin and Vit C seems to protect against oxidative stress by enhancing regeneration of endogenous antioxidants such as glutathione peroxidase, which might reduce deleterious effects of ROS [51]. This would suggest that the combination might stimulate synergistic response greater than individual antioxidant.

Recent meta-analysis showed that GGT activity is strongly associated with CVD risk in the general population [52]. Moreover, a longitudinal increase in GGT, independent of baseline level and even within its normal range, significantly increased risk of fatal CVD [53]. Therefore, reduction of GGT may reflect the reduction of CVD risk. In this study, the level of GGT was within normal range at baseline in all participants, but a greater reduction of GGT level was found in the VMP group compared to the PLA group. It is interesting to note that subjects in the VMP group with higher baseline GGT values showed a significantly more pronounced decrease in their GGT values as compared to the PLA group subjects with higher baseline GGT values.

The limitation of our study is the short duration of the intervention and unbalanced gender ratio; therefore, we can use only surrogate markers of CVD risk. Two large RCT in which total mortality and CVD mortality were used as outcomes did not confirm any influence of long-term (11 and 7.5 years) use of multivitamin or antioxidant supplements on the number of CVD events or CVD related mortality [54,55]. However, blood levels of vitamins and antioxidants were not controlled in these studies and the compliance was accessed through questionnaire and telephone surveys. Moreover, in one of the studies, only men were enrolled; therefore, gender analysis of the results was not possible. On the contrary, a retrospective epidemiological study showed that use of multivitamin-mineral supplements for ≥3 years was associated with reduced risk of CVD mortality among women when models controlled for age, race, education, body mass index, alcohol, aspirin use, serum lipids, blood pressure and blood glucose/glycated hemoglobin [56]. These findings were confirmed by the results of a Swedish study in which the use of multivitamins was associated with reduced risk of myocardial infarction, and this reduction was even greater if women used supplements for ≥5 years [57]. Our study demonstrated that short-term use of VMP leads to changes in circulating antioxidants and biomarkers of CVD risk in a Russian population. However, to confirm whether these effects translate into reduced CVD mortality, large, long-term controlled studies are needed.

## 5. Conclusions

Dietary VMP supplementation improved the nutrient status and reduced biomarkers of heart health risk (Hcy and GGT) in a Russian population.

## Figures and Tables

**Table 1:** Study inclusion and exclusion criteria.

**Inclusion Criteria:**
Generally healthy men and women aged from 40 to 70 years of age preferable smokers and who regularly consumes alcohol, and consuming fewer than 12 items found on the Recommended Foods Checklist (see below) per week.
Individual should be judged to be in good general health based on an interview and abbreviated physical exam.
Individual understands the procedures and agrees to participate in the study.
Individual is able and willing to provide written informed consent and confidentiality agreement.
**Exclusion Criteria:**
Use of dietary supplements within one week of Day 1. Supplements include any vitamins, minerals, and herbal products, including herbal drinks.
Presence of cardiovascular disease, hypercholesterolemia, cancer, diabetes mellitus, or any other chronic health condition identified from the findings of the interview.
Currently treated for uncontrolled hypertension or blood pressure greater >140 mm Hg systolic or >90 mm Hg diastolic during seated, resting measurement on two consecutive occasions during visit 1.
Therapeutic uses of coumadin, aspirin, or other medications that influence hemostasis within four weeks of Day 1.
Participation in another clinical trial within 30 days of enrollment into the study.
History or current abuse of drugs or alcohol, or intake >4 alcoholic beverages per day.
Known hypersensitivity to study product or any ingredient in study product.
A change in hormone therapy, including oral contraceptives, within 4 weeks prior to screening, or unwilling to maintain current hormone therapy/oral contraceptive use throughout the course of the study.
Pregnant or lactating women, or pre-menopausal women not using a medically approved form of birth control.
Any condition that the Principal Investigators believe may put the subject at undue risk.

**Table 2:** Summary of study time line and measurements.

Study Measurements	Office Visit 1	Office Visit 2	Office Visit 3	Office Visit 4	Office Visit 5	Office Visit 6	Phone Interview
	Day 0	Day 1	Day 14	Day 28	Day 42	Day 56	Day 57–60
Informed Consent	X						
Pregnancy test (urine)	X						
Recommended Foods Checklist	X					X	
Physical Exam	X					X	
Medical History, Family Medical History, Dietary Behavior and Lifestyle Questionnaires	X					X	
Food Frequency Questionnaire	X					X	
Blood lipid profile determination	X					X	
Blood concentrations of selected nutrients determined	X			X		X	
Biomarkers of Heart health risk determined	X					X	
Safety measurements (Serum Chemistry Panel, Hematology, and Urinalysis)	X						
Adverse Events Questionnaire		X	X	X	X	X	X
Inform subject’s eligibility		X					
Follow-up as Good Clinical Practice							X
Dispense the product		X		X			
Collect the product			X	X	X	X	

X–performed.

**Table 3:** Subjects baseline characteristics.

Variable	PLA Group (*n* = 60)	VMP Group (*n* = 60)	*p*-Value
Male/Female (*n*)	9/51	12/48	0.630
Age (year)	48.9 ± 7.6	49.5 ± 7.6	0.660
Smokers, (*n*) (%)	14 (23%)	16 (27%)	0.830
Alcohol consumers, (*n*) (%)	39 (65%)	30 (50%)	0.140
Physically active, (*n*) (%)	26 (43%)	22 (36%)	0.580
**Anthropomorphic Measurement**			
Weight (kg)	74.27 ± 15.94	75.72 ± 15.47	0.615
BMI (kg/m^2^)	26.58 ± 5.72	26.89 ± 4.92	0.739
Waist/Hip ratio	0.87 ± 0.13	0.88 ± 0.15	0.516
Systolic blood pressure (mmHg)	116.30 ± 9.05	117.83 ± 9.76	0.374
Diastolic blood pressure (mmHg)	75.08 ± 8.49	75.58 ± 6.89	0.724
**Heart Health Biomarkers**			
Homocysteine (µmol/L)	15.98 ± 6.62	18.55 ± 10.53	0.112
hs-CRP (ng/mL)	1922 ± 1937	2551 ± 2594	0.135
Oxidized LDL (ng/mL)	3939 ± 4109	3546 ± 3011	0.552
GGT (U/L)	22.41 ± 12.62	26.18 ± 18.71	0.199
Uric acid (µmol/L)	236 ± 83	241 ± 68	0.725
Total Cholesterol (mmol/L)	5.13 ± 0.93	5.10 ± 1.02	0.863
HDL cholesterol (mmol/L)	1.61 ± 0.46	1.57 ± 0.41	0.571
LDL cholesterol (mmol/L)	3.37 ± 0.76	3.29 ± 0.80	0.590
Triglycerides (mmol/L)	1.04 ± 0.58	1.06 ± 0.66	0.818

Values expressed as mean ± S.D. BMI = body mass index; PLA = placebo; multivitamin, VMP = multimineral and phytonutrient; hs-CRP = high-sensitivity C-reactive protein; LDL = low-density lipoprotein; GGT = gamma-glutamyl transferase; HDL = high-density lipoprotein.

**Table 4:** Baseline dietary intake from food frequency questionnaire and blood nutrient levels.

Variable	PLA Group	VMP Group	*p*-Value
RFS	8.8 ± 1.3	8.8 ± 1.3	0.943
Calories (kcal/day)	2449 ± 759	2481 ± 828	0.825
Protein (g/day)	87 ± 31	85 ± 37	0.712
Carbohydrate (g/day)	220 ± 100	244 ± 130	0.252
Sugar (g/day)	64 ± 52	74 ± 69	0.352
Fat (g/day)	117 ± 44	117 ± 47	0.999
Saturated fat (g/day)	39 ± 16	39 ± 14	0.842
Polyunsaturated fat (g/day)	28 ± 15	27 ± 15	0.800
ω-6 (g/day)	25 ± 14	25 ± 14	0.936
ω-3 (g/day)	3.2 ± 1.5	3.0 ± 1.6	0.544
Cholesterol (mg/day)	266 ± 137	281 ± 208	0.636
Na (mg/day)	3888 ± 1532	3805 ± 1660	0.777
K (mg/day)	3643 ± 1709	3515 ± 1691	0.682
Ca (mg/day)	1137 ± 480	1147 ± 515	0.913
P (mg/day)	1520 ± 496	1512 ± 575	0.929
Mg (mg/day)	378 ± 144	378 ± 167	0.986
Fe (mg/day)	17.8 ± 8.6	17.61 ± 9.77	0.893
Vit A (mg/day)	0.34 ± 0.24	0.42 ± 0.24	0.090
Vit B1 (mg/day)	1.03 ± 0.42	1.06 ± 0.55	0.720
Vit B2 (mg/day)	1.57 ± 0.56	1.61 ± 0.70	0.736
Niacin (mg/day)	15.0 ± 6.8	14.7 ± 7.4	0.821
Vit C (mg/day)	191 ± 147	187 ± 137	0.874
**Blood Nutrients**			
β-Carotene (µg/dL)	138 ± 66	133 ± 54	0.662
Total tocopherols (mg/dL)	1.88 ± 0.43	1.87 ± 0.48	0.872
Vit C (mg/dL)	11.76 ± 8.68	9.66 ± 8.07	0.173
RBC Folate (µg/L)	7.24 ± 2.98	7.10 ± 3.04	0.798
Vit B6 (µg/L)	12.7 ± 5.1	11.6 ± 5.2	0.277
Vit B12 (ng/mL)	332 ± 174	339 ± 215	0.848
Selenium (µg/L)	96.2 ± 9.3	98.6 ± 9.1	0.158
Zinc (µmol/L)	15.0 ± 3.0	15.7 ± 2.9	0.159
Magnesium (mmol/L)	15.0 ± 8.7	15.0 ± 8.7	0.786
Quercetin (ng/mL)	13.9 ± 7.0	13.4 ± 8.0	0.727

Values expressed as mean ± S.D. RFS = recommended food score.

**Table 5:** Change in blood nutrient status after 28 and 56 days of dietary supplementation.

Blood Nutrients	Intervention Period	PLA Group	VMP Group	*p*-Value ‡
β-Carotene (µg/dL)	Day 1–Day 28	11.02 ± 41.44 *	6.90 ± 51.87	0.684
	Day 1–Day 56	0.00 ± 54.70	0.24 ± 56.07	0.491
Total tocopherol (mg/dL)	Day 1–Day 28	−0.07 ± 0.53	0.06 ± 0.63	0.112
	Day 1–Day 56	−0.06 ± 0.55	−0.01 ± 0.57	0.324
Vit C (mg/dL)	Day 1–Day 28	0.36 ± 10.28	−0.99 ± 8.76	0.780
	Day 1–Day 56	2.97 ± 9.09	8.21 ± 9.27	0.001
RBC Folate (µg/L)	Day 1–Day 28	0.71 ± 5.86	3.40 ± 5.88 *	0.007
	Day 1–Day 56	−0.23 ± 6.01	3.20 ± 7.18	0.003
Vit B6 (µg/L)	Day 1–Day 28	−4.58 ± 6.48 *	−0.02 ± 7.26	0.0002
	Day 1–Day 56	−6.83 ± 7.58 *	−2.46 ± 7.87	0.001
Vit B12 (ng/mL)	Day 1–Day 28	−90 ± 193 *	−87 ± 227 *	0.470
	Day 1–Day 56	−84 ± 206 *	−39 ± 239	0.136
Zinc (µmol/L)	Day 1–Day 28	0.49 ± 2.70	−0.45 ± 2.30	0.978
	Day 1–Day 56	−0.11 ± 2.96	−0.72 ± 2.84	0.872
Selenium (µg/L)	Day 1–Day 28	1.60 ± 9.07	1.30 ± 8.18	0.575
	Day 1–Day 56	1.23 ± 7.99	−0.40 ± 10.09	0.836
Quercetin (ng/mL)	Day 1–Day 56	−0.08 ± 9.45	4.28 ± 8.58 *	0.005

Values expressed as mean ± S.D. ‡ Between group comparison. * Within group comparison (*p* < 0.05).

**Table 6:** Change in CVD biomarkers after 56 days of dietary supplementation.

Heart Health Biomarkers	PLA Group	VMP Group	*p*-Value ‡
Hcy (µmol/L)	−0.82 ± 8.16	−3.97 ± 10.09 *	0.031
hs-CRP (ng/mL)	515 ± 2642	134 ± 3507	0.251
Oxidized LDL (ng/mL)	1821 ± 4172 *	1907 ± 4264 *	0.544
GGT (U/L)	1.99 ± 9.86	−1.68 ± 14.53 *	0.054
Uric acid (µmol/L)	2.45 ± 50.37	−7.52 ± 62.20	0.168
Total cholesterol (mmol/L)	−0.18 ± 0.93	−0.16 ± 0.82	0.563
HDL cholesterol (mmol/L)	−0.08 ± 0.34	−0.04 ± 0.33	0.232
LDL cholesterol (mmol/L)	−0.17 ± 0.79	−0.12 ± 0.76	0.625
Triglycerides (mmol/L)	0.10 ± 0.42	0.02 ± 0.44	0.159

Values expressed as mean ± S.D. ‡ Between group comparison. * Within group comparison (*p* < 0.05); CVD = cardiovascular disease.

## Appendix A

The list of 23 foods used to calculate the RFS. One point is given for each item below if subjects recalled eating them at least once a week.

Apples/pears; Baked or broiled fish; Baked or stewed chicken or turkey; Baked or stewed chicken or turkey; Black currants; Broccoli; Carrots or mixed vegetables with carrots; Cooked cereals; Dark bread; Grapefruit; Green salad; High fiber/bran/granola cereals; Marrow; Orange/grapefruit juice; Oranges; Other fruit juices; Paprica; Potato; 2% milk; Skim or 1% milk; Spinach; Spring (sprout) onion; Tomatoes; White cabbage.
